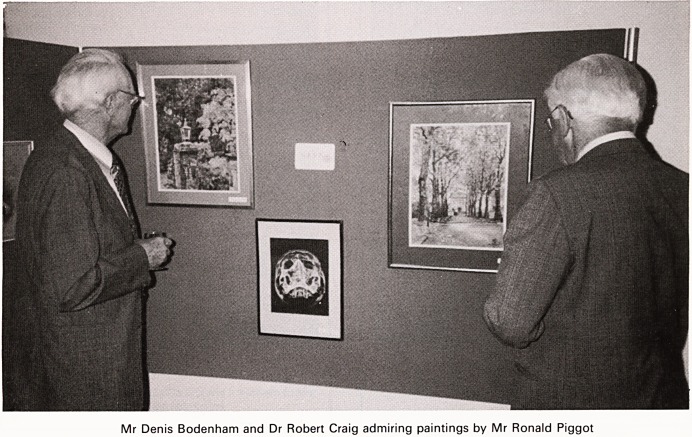# Exhibition of Art and Craft Work by Bristol Doctors

**Published:** 1985-07

**Authors:** 


### Exhibition of Art and Craft Work by Bristol Doctors

The third Exhibition of Art and Craft Work by Bristol doctors 'and their relatives' organised by the Royal Medical Benevolent Fund was on view at the Southmead Hospital Postgraduate Centre on Sunday, 9th June and was followed by a buffet supper. The first such exhibition was in 1980 and the second in 1982, each has surpassed the last in number and variety of exhibits, this time 45 exhibitors sent in their work. Some works were on sale on commission, this with sale of tickets and a raffle produced a profit of £700 for the fund and on this account alone it must be accounted a success. There were some beautiful things in the exhibition and it had been very 'professionally' set up but for two other reasons the exhibition was especially enjoyable and most unusual. First, nearly everything had been done just for the joy of it and with no thought of exhibiting and secondly, though nearly everyone knew, or knew of every exhibitor, it so often came as a complete surprise to find that he or she pursued such hobbies. Of course a few of the exhibitors were well known and experienced artists whose works regularly appear in our local galleries – Dr. Pamela Harman, Mrs. Phoebe Tulloh, Mrs. K. C. P. Smith and Mr. Gary James and one or two others like Mr. Ron Piggott and Mr. John Crossley are well known for their expert landscapes. One expected to see and enjoy their work, but one hardly expected to see so much else that was so good, particularly when one had known the artist for years and never knew he or she had ever handled a brush or a chisel or a potter's wheel. If there is a criticism it is that there was not enough time to see everything properly between the opening of the exhibition and the serving of supper and after supper it was speedily dismantled. For this reason your correspondent does not feel able to offer detailed comment on the works shown but feels that a list of the artists and their work will be of interest. In addition to the exhibitors named above there were paintings by Dr. Susanne Clarke, Dr. Sue Heaton, Mrs. Pamela Holford, Mr. Robert Horton, Miss Kamatt, Dr. Michael Lennard, Mrs. Barbara Shepherd, Dr. Stewart Silvey, Mrs. Muriel Thwaites, Mr. Michael Wilson. Book binding by Dr. John Burton, Upholstery by Mrs. Shirley Celestin, furniture by Dr. Bill Barritt, an appliquéd wall hanging

Mr Denis Bodenham and Dr Robert Craig admiring paintings by Mr Ronald Piggot by Lady Middlemiss and a bust of her father by Celia Middlemiss, a display cabinet by Dr. Joe Mandeville. Dr. Marie Freeman showed dolls and embroidery, Mrs. Susan Walker demonstrated lacemaking, Mrs. Bozana Easty showed crochet work, Mrs. Clive Johnson and Mrs. Barbara Shepherd showed embroidery, Mrs. Liz Dieppe showed lace coasters and Dr. Vivienne Zinober knitting. There was wood carving by the late Dr. Colin Tribe. Ceramics by Mr. Peter Witherow and his daughter, by Mrs. Charles Ward and by Dr. Joffe. There was sculpture by Dr. Pamela Harman, glass engraving by Dr. J. Owen and stained glass by Mrs. Caroline Whitwell. Mrs. MacCormack showed silver jewelry and Dr. Rajid Mal Bonsai trees. There were photographs by Dr. David Felce, Mr. Roger Feneley, Dr. Cameron and Rose Kennedy, Dr. Tony Lavelle, Professor Matthew's children, Dr. Peter Simpson and Mr. Paul Stableforth. None of this would have been possible without the initiative and hard work of the organisers headed by their Chairman Mrs. Rosemary Mulvein, their Secretary Mrs. Susan Silvey and their Treasurer Mrs. Dinah Bernard with the assistance of their Committee Mrs. Jenny Harvey, Dr. Susan Heaton, Mrs. Paddy Jelen, Dr. Gwyneth Jephcott, Mrs. Rachel Maxwell, Mrs. Caroline Paine, Mrs. Judy Torrens and Mrs. Judy Williamson.

## Figures and Tables

**Figure f1:**